# Cost-effectiveness evaluation of bovine tuberculosis surveillance in wildlife in France (Sylvatub system) using scenario trees

**DOI:** 10.1371/journal.pone.0183126

**Published:** 2017-08-11

**Authors:** Julie Rivière, Yann Le Strat, Pascal Hendrikx, Barbara Dufour

**Affiliations:** 1 Ecole vétérinaire d’Alfort (Alfort National Veterinary School), Research unit EpiMAI USC Anses (Epidemiology of Animal Infectious Disease), Université Paris Est, Maisons-Alfort, France; 2 Santé publique France, French national public health agency, Saint-Maurice, France; 3 Agence nationale de sécurité alimentaire nationale, French Agency for Food, Environmental and Occupational Health and Safety (Anses), Unit of coordination and support to surveillance, Maisons-Alfort, France; Universidade de Aveiro, PORTUGAL

## Abstract

Bovine tuberculosis (bTB) is a common disease in cattle and wildlife, with health, zoonotic and economic implications. Infected wild animals, and particularly reservoirs, could hinder eradication of bTB from cattle populations, which could have an important impact on international cattle trade. Therefore, surveillance of bTB in wildlife is of particular importance to better understand the epidemiological role of wild species and to adapt the control measures. In France, a bTB surveillance system for free-ranging wildlife, the Sylvatub system, has been implemented since 2011. It relies on three surveillance components (SSCs) (passive surveillance on hunted animals (EC-SSC), passive surveillance on dead or dying animals (SAGIR-SSC) and active surveillance (PSURV-SSC)). The effectiveness of the Sylvatub system was previously assessed, through the estimation of its sensitivity (*i*.*e*. the probability of detecting at least one case of bTB infection by each SSC, specie and risk-level area). However, to globally assess the performance of a surveillance system, the measure of its sensitivity is not sufficient, as other factors such as economic or socio-economic factors could influence the effectiveness. We report here an estimation of the costs of the surveillance activities of the Sylvatub system, and of the cost-effectiveness of each surveillance component, by specie and risk-level, based on scenario tree modelling with the same tree structure as used for the sensitivity evaluation. The cost-effectiveness of the Sylvatub surveillance is better in higher-risk departments, due in particular to the higher probability of detecting the infection (sensitivity). Moreover, EC-SSC, which has the highest unit cost, is more efficient than the surveillance enhanced by the SAGIR-SSC, due to its better sensitivity. The calculation of the cost-effectiveness ratio shows that PSURV-SSC remains the most cost-effective surveillance component of the Sylvatub system, despite its high cost in terms of coordination, sample collection and laboratory analysis.

## 1. Introduction

Bovine tuberculosis (bTB) is a chronic disease caused by *Mycobacterium bovis* or less frequently by *M*. *caprae*, which affects livestock, companion animals, wild species and humans [1]. The European Commission has considered France to be bTB-free since 2000, but infected herds and cases in wildlife (red deer–*Cervus elaphus*–, roe deer–*Capreolus capreolus–*, Eurasian wild boar–*Sus scrofa*–and badgers–*Meles meles–*) are still detected each year in some areas [2]. Wild species, and particularly maintenance hosts, represent an obstacle to the eradication of bTB in cattle, as potentially source of re-infection [3, 4, 5, 6]. Furthermore, the bTB transmission between wildlife species has a potential impact on biodiversity, ecotourism and commercial game farming [7] and the control measures of bTB in wildlife could be difficult to implement and maintain in such multi-host systems especially when species share habitat with cattle (e.g. control of population densities through culling on wild boars, deer, …) [8, 9].

A national surveillance system for bTB in wildlife, the Sylvatub system, was launched in France since 2011. Its aims are the early detection of cases and the monitoring of infection levels in affected areas. The Sylvatub system relies on three independent surveillance system components (SSCs): passive scanning surveillance on hunted wild species, passive surveillance on dead or dying wild animals and planned active surveillance on hunted or trapped wild animals. As bTB surveillance in wildlife species is subject to several constraints, the effectiveness of the three SSC of the Sylvatub system was assessed previously, for each SSC, specie and risk-level [10].

However, the evaluation of surveillance activities cannot be based alone on their ability to detect cases (effectiveness). The costs of surveillance must also be considered, due to limited financial resources facing an increasing need for surveillance and control programmes in animal health. It is therefore essential to develop tools to identify optimal strategies in terms of efficiency [11, 12, 13]. In developed countries, bTB induces major economic losses in the livestock sector, with costs to the cattle industry and government due to surveillance expenses (testing costs), and control measures (movement restrictions on the international trade of animals and their products and compensation for slaughtered cattle). While the cost of bTB in cattle has been widely studied in recent years [14] the economic aspects of the bTB infection in wildlife have rarely been investigated. Furthermore, such studies have mainly focused on the costs of control measures in wildlife species (for example, evaluation of the strategies’ costs for depopulation of wildlife reservoirs to stop the spread of bTB in cattle [15, 16, 17]), but rarely on the costs of surveillance activities. However, economic analyses are essential to justify the allocation of funds for surveillance, eradication and control programmes of animal diseases, and constitute a helpful tool to support decision in the choice of various strategies.

The objective of this study was the assessment, for the first time in France, of the costs and the cost-effectiveness of the bTB Sylvatub surveillance system in free-ranging wildlife in France. We used the scenario tree modelling approach, with the same model structure as for the efficiency assessment [10]. This method presents the advantage of combining several data sources based on non-probabilistic sampling, evaluating separately or together the effectiveness and the costs of several components in order to estimate the efficiency of surveillance strategies, all within the French context in terms of geography, resources and infrastructure [18, 19].

## 2. Materials and methods

### 2.1. Ethics statement

This study did not involve the deliberate killing of animals for the sole purpose of the study, as all samples were collected from animals trapped or hunted legally during the hunting season with appropriate permits, shot legally because of severe debilitation or found dead. All the samples included in this study were obtained from animals analyzed within an official context relating to bTB surveillance in free-ranging wildlife. All sampling procedures complied with national and European regulations and no specific ethics approval was therefore required.

### 2.2. Scenario trees and model description

The French surveillance system for bTB in wildlife consists of three independent SSCs: (1) passive surveillance on hunted wild boar, red deer and roe deer (EC-SSC); (2) passive surveillance on dead or dying animals for wild boar, red deer, roe deer and badger (SAGIR-SSC); (3) planned active surveillance on hunted wild boars, red deer and trapped badgers (PSURV-SSC). These SSCs are applied according to geographic risk, which is assessed on the basis of outbreaks in cattle or wildlife. Three levels of risk have been defined (low-risk, medium-risk, high-risk areas) and are regularly re-evaluated, depending on the evolution of the epidemiological situation in cattle and wildlife. The Sylvatub system has been described in detail in [10]; a brief description of the functioning of each SSC is given in following paragraphs (2.3.2, 2.3.3, 2.3.4). The effectiveness of the Sylvatub system was evaluated with a scenario tree modelling method assessing the sensitivity of detection, as the probability of detecting at least one animal positive for bTB for a given prevalence in the population, by SSC, specie and risk level [10]. A tree represents all the events influencing the detection of the infection as nodes dividing the population into groups of animals with similar probabilities of being infected and detected [18]. Each of these category nodes may have one or more possible outcomes, with a specific probability of occurrence estimated from historical data, published findings or expert opinion.

In order to identify the most cost-effective SSC or SSCs combination of the Sylvatub system, it was necessary to relate the costs with the achieved sensitivity, either at the individual or collective level. Therefore, we first performed a simple cost assessment for each SSC for one year, and then we calculated the cost-effectiveness ratio for each SSC, by specie and level of risk. We used the same structure and content (nodes and epidemiological input parameters) of the scenario trees as for the effectiveness evaluation [10] (S1 Fig, S2 Fig, S3 Fig respectively for the EC-SSC, SAGIR-SSC and PSURV-SSC scenario trees structures).

### 2.3. Costs estimation per SSC

Economic data were included in scenario trees, for each step of the detection process. Mean costs were estimated for the 2013–2014 hunting season, by SSC, risk-level and specie, for one year. Cost variability was also estimated, as the importance of extreme values and their probability of occurrence may influence the choice of a surveillance strategy and thus provide information as objective as possible to decision makers. The estimated costs were divided into three main categories, which cover several costs:

- **Coordination**: organization of surveillance activities, meetings, communication (letters, convening letters, information, results…), backstopping and follow-up of the actors in their field missions, administrative and financial follow-up of the surveillance activities, salary of the stakeholders in charge of local and national coordination, etc. At the national level, coordination of the Sylvatub system is carried out by a coordinator, who organizes regular meetings at the national and local levels, defines the sampling protocols for the three SSCs and analyses the surveillance results. The cost of the national coordination was estimated by the coordinator himself, in terms of time spent per year for the coordination for each SSC and risk level (the daily fees were estimated at 300 € / day, including personal, structural and travel costs). At the local level, the Sylvatub system coordination is endorsed by various stakeholders for each SSC. Local conventions were used as economic data sources when available (for PSURV-SSC especially), otherwise, estimation of the time spent to the coordination activities was asked to the relevant stakeholders (with a daily fee at 200 or 300 € / day according to their status).
- **Collection** of animals, carcasses or samples: sampling and trapping equipment, compensation of actors for the sample collection, travels, storage and transport of carcasses or specimens to a laboratory. The collection costs are variable between SSCs, species and risk-levels (only for the PSURV-SSC for the latter), as they vary according to the collection protocol.
- **Laboratory analysis:** necropsy, sampling, bTB analysis in an accredited local laboratory (systematic culture, PCR if tuberculosis like lesion(s) (TBL(s)) are detected during necropsy), bTB confirmation analysis in the French national reference laboratory (NRL) if a non-negative result is obtained in a local laboratory, and transport of samples between laboratories. The costs of laboratory analysis are common to the various SSCs.

The further paragraphs present the collection of economic data and the method of costs estimation for each of these categories, by SSC, specie and risk-level if relevant. Some of the estimated costs present a certain variability (variations between species and/or areas, due to biological or organizational variation between departments or level of risk), some other a certain uncertainty (lack of knowledge, economic data not precisely known) and finally some both variability and uncertainty. To take this into account, law distributions were used to model economic data in scenarios trees.

#### 2.3.1. Laboratory costs for bTB analysis (common costs to the three SSCs)

For each animal, a necropsy is carried out for the detection of TBLs. The diagnostic process differs between SSCs: for EC-SSC at all risk levels and for SAGIR-SSC in low-risk areas, samples are analysed by culture and PCR only if TBLs are detected by the laboratory staff; whereas for SAGIR-SSC and PSURV-SSC in medium- and high-risk areas, the samples are systematically analyzed by culture, and PCR is performed on the TBLs, if present. If a non-negative result is obtained at a local laboratory, biological material is sent to the NRL for confirmation.

The average unit cost for the "laboratory analysis" category vary according to several factors:

- accreditation of the local laboratory for bTB tests (when it is not accredited, samples should be sent to an accredited laboratory, which leads to additional conditioning and transport costs): the proportion of accredited laboratories for bTB analysis in each risk level for the 2013–2014 season (10%, 30% and 40% respectively in low-, medium- and high-risk areas) was used to weight the estimated costs for each category and thus obtain an average unit cost per risk level area;
- presence or absence of TBL(s), impacting the realization of a PCR (which itself depends on the bTB infectious status, the specie and the age of the animal, estimated previously in [10]);
- number of analysis carried out by an accredited laboratory: the compensatory rate decreases if more than 100 analysis are carried out by the same laboratory;
- results of the bTB analysis obtained in the accredited local laboratory, impacting the realization of confirmatory analysis at the NRL (depending on the sensitivity and specificity of culture and PCR, estimated by experts);

The equipment used for the analysis and the salary of the technicians were not taken into account because they do not constitute a specific cost of the Sylvatub surveillance. As the number of bTB analyses carried out by each local laboratory was variable, unit laboratory costs were modeled by Pert distributions (Table 1).

**Table 1:** Costs estimation for the bTB laboratory analysis (common costs to the three SSCs).

Surveillance activity at laboratory	Unit cost (in euros, for one animal)
Necropsy	37
Sample collection and conditioning	16
Transport of samples to an accredited local laboratory	22
Culture in an accredited local laboratory	• 85 (if ≤ 100 cultures per year) • 42 (if > 100 cultures per year)
PCR in an accredited local laboratory	• 80 (if ≤ 100 PCR per year) • 60 (if > 100 PCR per year)
Transport of DNA extracts to the NRL	22
Transport of bacterial strains to the NRL	45
PCR in the NRL	15
Typing in the NRL	85
**Total cost of analyses in a local laboratory**	
• Non-accredited local laboratory	
○ Without TBL (culture only) ○ With TBL(s) (culture and PCR)	○ Pert (117; 140; 160) ○ Pert (177; 197; 210)
• Accredited local laboratory	
○ Without TBL (culture only) ○ With TBL(s) (culture and PCR)	○ Pert (79; 100; 122) ○Pert (139; 159; 202)
**Total cost of analyses in the NRL**	Pert (37; 130; 167)

#### 2.3.2. Specific costs for the passive surveillance by carcass examination (EC-SSC)

This surveillance component concerns hunted wild boar, red deer and roe deer. It is based on *post-mortem* examination and the voluntary submission by hunters (free of charge) of carcasses with macroscopic TBLs, in all geographic areas (*i*.*e*. regardless the local risk). The main detection nodes influencing the probability of bTB detection by the EC-SSC are the presence of macroscopic TBLs (modeled by a Pert distribution according to an expert panel as a function of the infectious status, the species and the age of the animal), and the detection of these TBLs by a hunter (which depends on the awareness of hunters–training and experience- and the species hunted), who subsequently reported his suspicions, triggering the diagnostic process [10]. For EC-SSC, the following expenses per animal were considered: the coordination costs at the national level by the coordinator and the national hunting federation (FNC), the collection cost of a suspected carcass by a hunter and its transport to a local laboratory, and the bTB analysis (Table 2).

**Table 2:** Costs estimations of the surveillance activities of each SSC of the Sylvatub system.

Surveillance activity	Unit cost (in euros, for one animal)
**Specific costs for the passive surveillance by carcass examination (EC-SSC)**	
National coordination activities by the FNC	Whatever the risk level: Pert (120; 144; 160)
National coordination activities by the coordinator	Whatever the risk level: Pert (58; 65; 72)
Collection of suspected hunted animals (with macroscopic TBL(s))	Whatever the risk level: 100 €
**Specific costs for the passive surveillance on dead or dying animals (SAGIR-SSC)**	
Local coordination activities	Whatever the risk level: 0 €
National coordination activities by the coordinator	• Medium-risk level: Pert (7; 12; 24) • High-risk level: Pert (5; 8; 17)
Collection of dead or dying animals	Whatever the risk level: 108.35 €
**Specific costs for the active surveillance (PSURV-SSC)**	
Coordination activities (local and national level)	• Medium-risk level ○ Badgers: Pert (152; 191; 227) • High-risk level ○ Badgers: Pert (31; 54; 110) ○ Deer and wild boars: Pert (30; 45; 59)
Collection	• Medium-risk level ○ Badger: 142 € ○ High-risk level ○ Badger: Pert (52; 80; 94) ○ Deer and wild boars: Pert (7; 15; 20)

- **Coordination**: The coordination costs by the FNC at the national level were calculated according to an estimated working duration per risk level (daily fee of 300 € / day). This duration varies each year, with the evolution of the epidemiological situation and cannot be directly related to the number of suspicions by year, because the coordination activities concern both the management of the current yearly suspect cases and also communication to prepare the next surveillance season. To estimate the average national coordination cost by the FNC for one collected suspect animal, the average coordination duration over the last three hunting seasons (2012–2013, 2013–2014, 2014–2015) was related to the average number of suspect cases for these periods (on average 27 days, i.e. 7,900 € per year for 55 suspicions). Furthermore, the average unit cost of coordination by the national coordinator was estimated by the coordinator himself in average number of days devoted per year to the coordination of this SSC (daily fee of 300 € / day). The number of days devoted to the EC-SSC was estimated at about 12 days (i.e. 3,600 € per year), for 55 suspicions, which correspond to an average unit cost of 65 € per suspect animal. According to the coordinator, the global coordination costs seems to be higher in medium- and high-risk level departments, but the number of suspicions is also higher in these areas (about 10, 25 and 20 suspicious cases on average per year in low-, medium- and high-risk level respectively): we thus considered that the unit cost is globally equivalent between the levels of risk. We finally used Pert distributions to model these average unit coordination costs, in order to take into account a certain variability and uncertainty in the working duration and in the number of suspicions per hunting season, respectively Pert (120; 144; 160) for the FNC coordination and Pert (58; 65; 72) for the coordinator coordination. There is no compensation of potential local animation costs for this SSC.
- **Collection**: A compensation of 100 € per suspect animal is paid to the local hunting federation, whatever the specie and the risk level, which covers the time spent for travels and the management of the suspicion. No additional compensation for consumables was foreseen, as hunters already have all the necessary equipment for the management of suspect cases.

#### 2.3.3. Specific costs for the passive surveillance on dead or dying animals (SAGIR-SSC)

This surveillance component relies on field stakeholders (hunters, local hunting federations and technicians from the National Hunting and Wildlife Office) providing an inventory of dead or moribund animals. In low-risk level, this SSC is independent of the Sylvatub system. In medium- and high-risk level areas, the SAGIR network receives assistance, free of charge (compensated by the French Ministry of Agriculture within the scope of Sylvatub) for the collection of animals and for systematic bTB analysis, even in the absence of TBL detection during necropsy. The detection of an infected wild animal by the SAGIR-SSC depends on the probability of a dead or moribund animal being collected by a stakeholder (estimated from expert opinion, depending on its size–species and age-, and the risk level), and on the probability for a wild animal to have TBLs detected at the laboratory (estimate from a multivariate logistic regression applied on Sylvatub data, adjusted on the infectious status, species and age class) [10]. The specific costs for the SAGIR-SSC in low-risk level were not estimated because it is independent of the Sylvatub system. Only the reinforced surveillance activities on wild boars, deer and badgers in medium- and high-risk level areas are compensated for Sylvatub and covers: the coordination, the collection of dead or dying animals, and laboratory costs (Table 2).

- **Coordination**: The costs of the national coordination were estimated by the coordinator to be on average 1,200 € per year for all medium- and high-risk level areas (about 4 days for all these departments, *i*.*e*. for 27 departments), which correspond, when reported to an average number of collected animals by this SSC and to take into account variability, to a modeled Pert (7; 12; 24) at medium-risk level and a Pert (5; 8; 17) at high risk-level. There is no compensation of potential local animation costs for this SSC.
- **Collection**: The unit cost for collecting a dead or dying animal was estimated at 108.35 € per animal (fixed compensation of 100 € per collected animal for the transport–kilometers and time spent–, and costs of consumables for the transport such as gloves, bags, etc., which were estimated at 8.35 € per collected animal).

#### 2.3.4. Specific costs for the active surveillance (PSURV-SSC)

For the PSURV-SSC, systematic bTB analysis are conducted on a planned sample of 15 badgers trapped within a radius of 1 km around bTB cattle outbreaks in medium-risk areas, and on samples of a hundred badgers and/or a hundred wild boars and/or about sixty red deer in larger areas within high-risk zones. This SSC depends on fewer factors, as a predetermined number of animals should be collected and analysed, even if no TBLs are detected: the main factor likely to influence the sensitivity of this SSC is therefore the diagnostic process [10]. The specific costs for the PSURV-SSC cover the organization and planning of surveillance campaigns, the coordination and local supervision of actors and laboratory costs (Table 2). They vary mainly from one department to another, because the practical organization is determined locally according to local characteristics and operational constraints. Calculated costs for the 2013–2014 hunting season for several departments of medium- and high-risk levels were used as example.

● **Coordination**: Coordination costs mainly concern meetings to organize and coordinate the trapping campaign, material preparation (purchase, preparation and distribution of collection kits).
- In medium-risk areas, the average unit cost of the local coordination was estimated at about 127 € per collected badger. For this estimation, the example of one medium-risk department was analysed in detail, in which 39 badgers were collected in 2013. Based on the administrative and financial report established by local stakeholders, the local coordination for trapping was estimated at 2,596 € (1,092 € for the participation to the meetings–hourly compensation rate of 16.82 €/h for 2 hours and 25 persons—in addition with 1,504 € for the supervision of trapping badgers–hourly compensation rate of 16.82 €/h for 79 hours and 0.32 €/km for 548 kilometers travelled), which represents a unit cost of 67 € for the local coordination for trapping badger. Furthermore, the coordination by the local responsible of the veterinary administrative service was estimated at 2,340 € (estimation of about 12 days devoted to this activity, for a daily fee of 200 €), i.e. 60 € per badger. Thus, the global local animation (administrative responsible for veterinary services and trapping responsible) was estimated at 127 € per badger collected. In addition, national coordination was estimated at 48 days for all medium-risk level departments (15 departments), *i*.*e*. approximately 3.2 days (960 €) per department. The average number of badgers collected in these departments varies between 10 and 30 depending on the number of cattle outbreaks, according to the previous surveillance campaigns (10 badgers when there is only one cattle outbreak and few badgers in a radius of 1 km around; sometimes 30 when there are several cattle outbreaks in the same medium-risk department). Thus, the unit cost of the national coordination was modeled by a Pert (25; 64; 100) in these areas. Thus, the unit cost for all coordination activities in medium-risk level (local and national level) was modeled by a Pert (152; 191; 227) (Table 2).
- In high-risk areas, the average unit costs of coordination (including local and national coordination) were modeled by a Pert (31; 54; 110) for badgers and Pert (30; 45; 59) for wild boars and red deer. For these estimation, three departments of high-risk level were studied, as the local organization could be extremely different between departments. The mean average time spent for the local coordination by the administrative responsible for veterinary services is about 144 days (i.e. 28,000 € for a daily fee of 200 € per day). In addition, an average on the compensation for meetings and supervision of collection activities was estimated for badger in one hand and for wild boars and deer in the other hand. These costs were reported to the number of animals collected in each of these departments. In addition, the national coordinator has estimated the time spent to this activity at 16 days for badgers and 16 days for wild boars and deer, for all high-risk levels (i.e. 4.800 € per year, for 10 departments). Reported to the mean collected animals in such areas per year, the unit cost for the national coordination was estimated at 3 € per animal, whatever the species. Thus, the unit cost for all coordination activities (local and national level) was modeled by a Pert (31; 54; 110) for badgers and by a Pert (30; 45; 59) for wild boars and red deer (Table 2).
● **Collection**: these costs include the time spent and distances traveled per actor, and the costs for materials. The unit cost for collecting badgers in medium-risk level areas was estimated from the administrative and financial report of the department choose for example, at 142 € per badger. In high-risk level areas, the collection costs were studied in three departments, including the material for the collection of badger and wild boar and deer, the time spent for the collect of animals (hourly compensation rate, travel compensations, compensation for collection, storage and transport to the local laboratory). The global financial compensation in each local convention were then reported to the number of animals collected in each department. Thus, the average unit compensation cost for collection activities was modeled in high-risk areas by a Pert (52; 80; 94) for badgers and Pert (7; 15; 20) for wild boars and red deer (the Pert represents the variability of situations between departments).

#### 2.3.5. Costs calculations

The costs of the Sylvatub system were estimated first at the animal level, based on (1) estimated average total unit cost for each category (Tables 1 and 2), (2) the type of analysis carried out in the local laboratories (culture, PCR according to the probability of presence of TBL detected at necropsy), (3) the proportion of accredited laboratories in each risk level and (4) the probability of bTB analysis performed at the NRL. The scenario trees were implemented in Excel and the Monte Carlo method was run stochastically with @RISK (Decision Tool, version 6). The structure and content (nodes and epidemiological input parameters) of the scenario trees were the same as for the effectiveness evaluation [10]. Thus, costs estimated here were combined with the probability of realization of each of the surveillance activities: for each SSC, specie and risk level, the mean with 95% confidence interval of the total unit costs were estimated from 10,000 simulated values, summing the costs involved in coordination, collection and laboratory analysis for one animal.

Then, average annual collective expected costs were estimated by Monte Carlo simulations for a "typical" department, for each risk level, based on (1) estimated average unit total costs by SSC, specie and risk level (Tables 1 and 2), and (2) an average expected number of animals collected, by SSC, specie and risk level, calculated from the mean of animal collected over the last two hunting seasons.

### 2.4 Cost-effectiveness

The scenario trees were developed and parameterized for each SSC, for which effectiveness and costs were estimated independently, in order to be able to compare the cost-effectiveness ratios of each component. Cost-effectiveness ratio is the cost of a surveillance system or a surveillance component divided by its sensitivity (as an attribute of its performance) [11].

First, the cost-effectiveness was estimated by Monte Carlo simulation for an infected animal, such that CEi = Cost_i / CSeU_i, where *i* represents the SSC, *CE* the cost-effectiveness for an infected animal, *Cost* the unit cost for an infected animal and *CSeU* the unit sensitivity [10, 11]. This allows to relate the cost to the probability of detecting the infection for an infected animal, for each component, risk level and wild specie. Lower the value of the ratio is, better is the cost-effectiveness of the surveillance activity.

In addition, the cost-effectiveness was also calculated at a collective scale, based on an average expected number of animals collected per SSC, risk level and specie, in order to estimate the overall cost in relation to the probability of detecting at least one bTB case in a department, and thus integrating the cost for all animals, including non-tuberculous animals that are also subject to surveillance.

## 3. Results

### 3.1 Costs estimations

#### 3.1.1. Unit costs

Table 3 shows the total unit costs estimated by Monte Carlo simulations for each SSC, risk level and specie, taking into account the three cost categories (coordination, collection, analysis). Costs are presented according to the infectious status of the animal, because this latter influences the probability that the animal has TBL(s) (and therefore the type of analysis performed in the local laboratory), and the probability of obtaining a positive or doubtful result (and therefore the need for confirmatory analysis at the NRL). We found that EC-SSC has the highest unit cost whatever the risk-level. The PSURV-SSC on badgers has also a high unit cost compared to the PSURV-SSC on wild boar and red deer, because of (1) higher coordination costs and (2) higher collection costs for this specie, due to the necessary equipment and the compensation of the actors for the collection of badgers, which is not the case for other species (wild boars and deer).

**Table 3:** Estimated total unit costs of surveillance activities of the Sylvatub system, by SSC, specie, risk-level and infectious status (in euros, mean [CI _95_%]).

SSC	Specie	Risk-level	bTB-infected animal	Not bTB-infected animal
EC-SSC	Deer and wild boar	Low	610 [560; 655]	510 [484; 538]
		Medium	603 [554; 645]	502 [476; 531]
		High	599 [550; 640]	498 [472; 527]
SAGIR-SSC	Badger	Medium	354 [318; 384]	248 [232; 263]
		High	346 [312; 375]	244 [228; 259]
	Wild boar	Medium	374 [336; 405]	250 [235; 265]
		High	365 [327; 396]	246 [231; 261]
	Red deer	Medium	359 [323; 389]	248 [233; 264]
		High	351 [315; 380]	244 [229; 260]
PSURV-SSC	Badger	Medium	566 [521; 606]	463 [432; 493]
		High	367 [322; 411]	264 [230; 301]
	Wild boar	High	311 [269; 348]	192 [169; 217]
	Red deer	High	218 [187; 250]	106 [89; 127]

#### 3.1.2. Total expected cost for one year (2013–2014 hunting season, 2014)

In order to estimate an average expected cost per department of a given risk level, we calculated the expected number of animals to be collected per SSC, specie and risk level as a mean of collected animals during the two last surveillance campaigns (Table 4). Thus, average annual expected costs were estimated by Monte Carlo simulations for a "typical" department for each risk level (Table 4), based on the estimated unit costs (Table 3) and the expected number of animals collected per SSC, specie and risk level. For one department of medium-risk level, whatever the specie, the average expected total cost of the Sylvatub surveillance was estimated as 13,430 € [4,745; 23,614]: EC-SSC would represent about 7% of this total cost, SAGIR-SSC about 18%, and the PSURV-SSC on badgers 75%. For one department of high-risk level, the average expected total cost was estimated at 89,165 € [34,162; 157,758]: EC-SSC would represent about 2% of the cost, SAGIR-SSC 5% and PSURV-SSC 93%. Taking into account the number of department of each risk-level, the total expected cost of the Sylvatub system for one year was estimated at 726,185€ [614,584; 843,224] (around 1% for low-risk level areas, 20% for medium-risk level areas and 79% for high-risk level areas). This total annual expected cost could be different from the real cost for one hunting season, considering the rate of achievement of the sampling for the PSURV-SSC (lower or higher than the number of animals that should be collected according to the regulatory text).

**Table 4:** Total expected annual cost for the Sylvatub system, by SSC, specie, risk-level and expected number of collected animals (in euros, mean [CI _95_%]).

SSC	Specie	Risk-level	Expected number of collected animal	Expected total cost (in euros)
EC-SSC	Red deer and wild boar	Low	Pert (0; 1; 3)	594 [125; 1,166]
		Medium	Pert (0; 2; 4)	1,006 [305; 1,718]
		High	Pert (0; 3; 6)	1,504 [455; 2,578]
SAGIR-SSC	Badger	Medium	Pert (0; 6 20)	1,852 [352; 3,781]
		High	Pert (0; 10; 60)	4,150 [485; 9,768]
	Wild boar	Medium	Pert (0; 1; 2)	258 [78; 439]
		High	Pert (0; 2; 4)	499 [146; 856]
	Red deer	Medium	Pert (0; 1; 2)	254 [76; 435]
		High	Pert (0; 2; 4)	289 [61; 565]
PSURV-SSC	Badger	Medium	Pert (5; 20; 45)	10,061 [3 934; 17,241]
		High	Pert (20; 150; 400)	45,574 [13,452; 85,371]
	Wild boar	High	Pert (70; 150; 300)	31,639 [17,385; 49,364]
	Red deer	High	Pert (10; 50; 100)	5,510 [2,178; 9,256]

### 3.2 Cost-effectiveness

#### 3.2.1. Unit cost-effectiveness ratio for an infected animal

**Fig 1:**
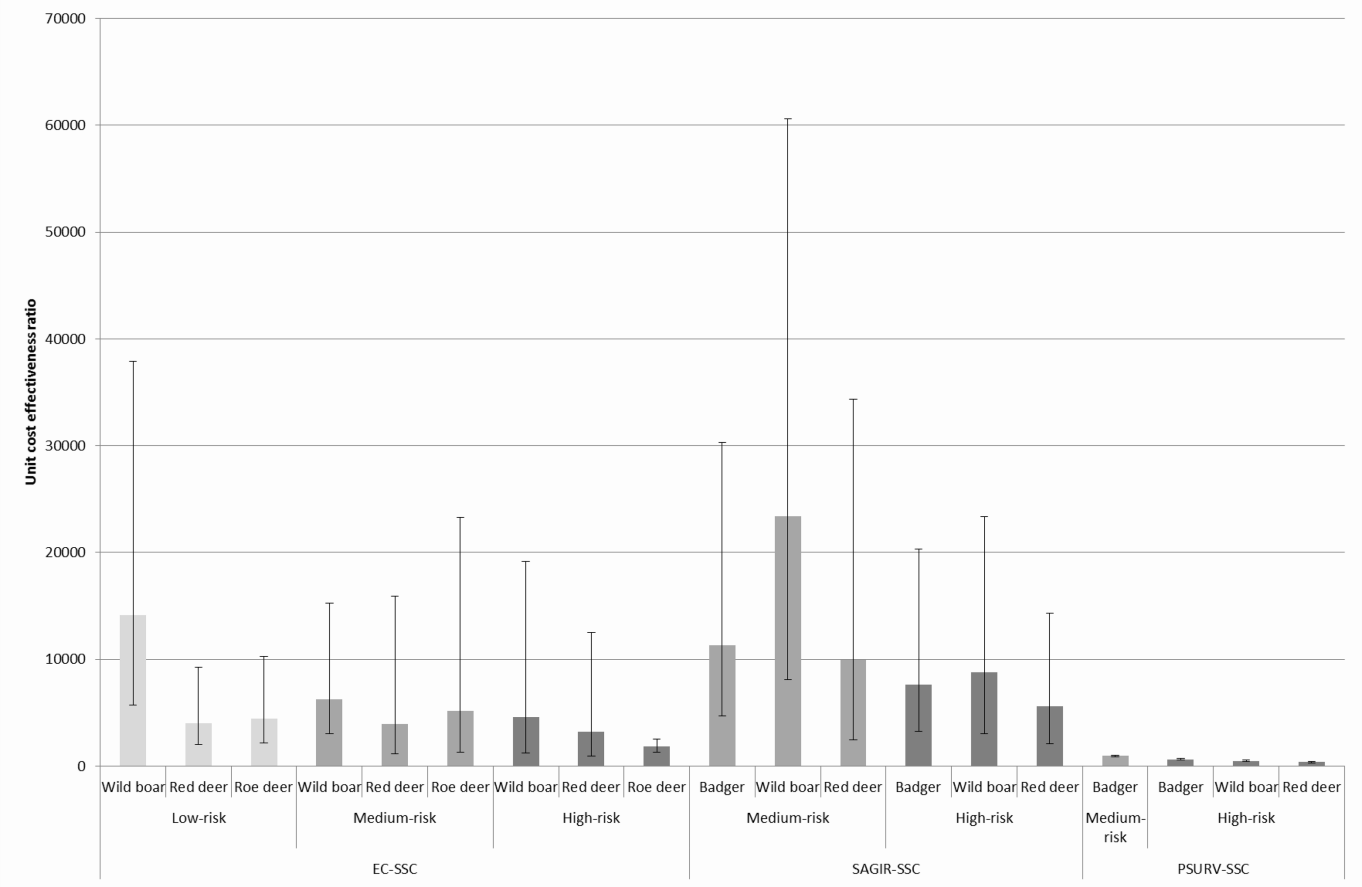
Unit cost-effectiveness ratios for an infected animal by SSC, risk-level and specie (mean [CI _95%_]).

The cost-effectiveness of detecting an infected animal was calculated (Fig 1), based on the cost for an infected animal (Table 3) and the individual sensitivity for an infected animal presented in [10], for each SSC, specie and risk level. The value of the cost-effectiveness ratio corresponds to the unit costs for the detection of an infected animal divided by the probability of detecting an infected animal. Thus, lower the value of the ratio is, better is the cost-effectiveness of the surveillance activity. Based on our results, PSURV-SSC is most cost-effective SSC but it is implemented only in high-risk level areas. Regarding the passive surveillance components, EC-SSC seems more efficient than SAGIR-SSC, due to the higher sensitivity of the EC-SSC, whatever the specie and the level of risk. The cost-effectiveness of EC-SSC is better in red deer than in wild boars, which can be explained by the fact that TBL(s) are more difficult to detect in these latter (lower sensitivity). If the hunter is not trained for lesion detection on carcasses, the SAGIR-SSC becomes more efficient than EC-SSC for wild boars in high-risk level areas and for deer in medium and high-risk level areas. The difference of cost-effectiveness between SSCs, species and risk-levels is however not important (overlap of confidence intervals) and these results could only be interpreted as tendencies.

#### 3.2.2. Collective cost-effectiveness ratio

**Fig 2:**
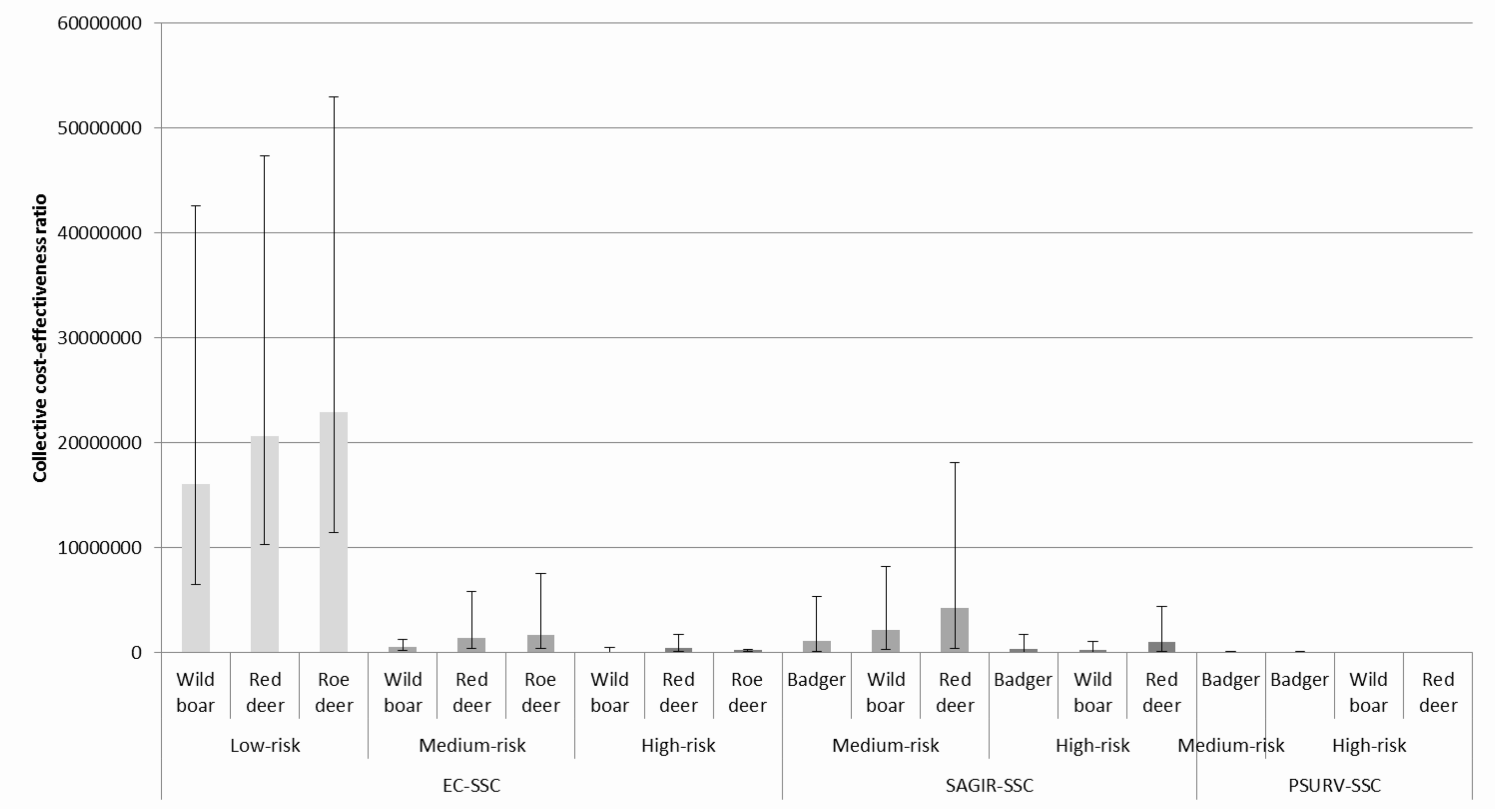
Collective cost-effectiveness ratio by SSC, risk level and specie, according to the design prevalence and an expected number of collected animals (mean [CI _95%_]).

The cost-effectiveness of surveillance by the Sylvatub system was also estimated at the collective scale (Fig 2), according to the expected number of animals collected per department (Table 4) and the design prevalence fixed by risk-level, specie and age class [10]. The results show that when prevalence is taken into account, EC-SSC is more cost-effective for wild boars than for deer and remains globally more efficient than SAGIR-SSC in the medium- and high-risk areas. Finally, PSURV-SSC remains the most cost-effective component of the Sylvatub system due to its high sensitivity, even if its collective costs are higher than other passive SSC. The cost-effectiveness is better in high-risk level areas, as the sensitivity is better than in low- and medium-risk level areas, considering that the design prevalence was set higher in these areas. The difference of cost-effectiveness between SSCs, species and risk-levels is however not important (overlap of confidence intervals) and these results could only be interpreted as tendencies.

## 4. Discussion

The surveillance of bTB in wildlife is of particular importance, as wild infected species could hinder the eradication of bTB from cattle population [3, 4] and as their role in the bTB epidemiology is sometimes unknown [20, 21]. The assessment of economic data of the Sylvatub surveillance system was realized for the first time and was particularly complicated, because of the heterogeneity of SSCs, of stakeholders and particularly of the practical organization between areas.

### 4.1 Costs estimations

We assessed variable and fixed costs supported by the French ministry of agriculture for each SSC and risk level for three main cost categories defined for the study (coordination, collection, laboratory analysis) for one year. To make a complete accurate analysis, further investigations should be conducted in order to assess the supplementary costs for hunters and local associations.

As investment costs were marginal, we only looked at operating costs. However, it might be worthwhile to explore the notions of investment and depreciation for certain equipment-related costs, such as the purchase of specific freezers for carcasses storage for example. Furthermore, even if the equipment used for the laboratory analysis and the salary of technicians are not specific costs of the Sylvatub system, they should be included in the part devoted to bTB as opportunity costs. The cost of hunter’s training has not been taken into account, as these training courses are not mandatory for the operation of the Sylvatub system. The costs of the usual operation of the SAGIR network in low-risk level areas and in deer for all areas were not included in this study because they are not specific to the Sylvatub system. However, it would be interesting to estimate the cost of this network related to bTB, in order to have a more comprehensive view of the overall cost of bTB surveillance in France. This implicates specific methodological analysis as the SAGIR network is generalist and concerns all the causes of mortality in wild animals.

Coordination costs vary according to the SSC: EC-SSC is mainly based on training, information and awareness-raising for voluntary actors, while PSURV-SSC relies on the organization and the preliminary planning of a surveillance campaign, and on the coordination and local supervision of the network of actors. It was particularly difficult to estimate an average unit cost representative of the PSURV-SSC, due to the heterogeneity of the local organization (collection of carcass by the local laboratory or delivery by actors, compensation for the collection of badgers, etc.). Furthermore, we assumed that the coordination costs were equivalent between species, but in reality they were certainly higher for badgers, due to the more complex organization of trapping and time spent for the management of compensation for trappers. Coordination and collection unit costs for PSURV-SSC are higher for medium-risk level areas than for high-risk level areas because the time spent (same compensation costs whatever the risk-level) is of the *pro rata* of a smaller number of animals collected (of the order of fifteen in medium-risk level, and several hundred in high-risk level).

Only costs related to bTB surveillance in wildlife were estimated in this study: the costs of control measures were not taken into account but were sometimes difficult to dissociate from those of surveillance. For example, only some of the badgers trapped within the 1 km radius of a cattle bTB outbreak are analyzed at the laboratory by the Sylvatub system, the other killed badgers are not submitted to bTB analysis but contribute to the regulation of the population.

### 4.2 Cost-effectiveness calculation

For the cost-effectiveness assessment, we used the stochastic scenario tree modelling approach described by Martin *et al*. [18], as it is well adapted to low expected disease prevalences and non-structured probabilistic sampling and allows to estimate both effectiveness and costs, either separately or together [22]. Scenario tree is a helpful structured and transparent tool to investigate performance and efficiency of various surveillance strategies and to provide evidence-based information to decision makers for the choice of the most cost-effective component.

Previous study [10] has shown that PSURV-SSC and EC-SSC had the highest collective sensitivity (CSe) for detecting at least one infected wild animal at the design prevalence, regardless of specie. The SAGIR-SSC had a very low CSe, whatever the specie considered, particularly at low (CSe < 1%) and medium (CSe < 5%) levels of risk. The reinforcement of the SAGIR network increases the sensitivity of this SSC, particularly at high levels of risk (CSe between 5% and 20%).

EC-SSC has a better sensitivity than SAGIR-SSC, but has a higher unit cost, in relation to the importance of coordination to maintain the awareness of hunters. According to our estimates, EC-SSC is the most costly on an individual estimation (Table 3), but not at a collective scale (Table 4). This can be explained by the high cost of coordination, given the low number of suspicions reported (on average about fifty per year). SAGIR-SSC is cheaper than EC-SSC on an individual scale (Table 3), as the cost of coordination is lower. However, it is more costly at a collective scale than EC-SSC because of the higher number of animals that are collected by this SSC (in particular badgers) (Table 4). However, the average cost-effectiveness ratio based on hunter training shows that surveillance enhanced by the SAGIR-SSC becomes more efficient than EC-SSC if the hunter is not trained for lesion detection on carcasses for wild boars in high-risk level areas and deer in medium- and high-risk areas. In higher risk areas, passive surveillance (EC-SSC and SAGIR-SSC) costs about 16 times lesser than PSURV-SSC, which is characterized by high costs, in terms of coordination, collection and laboratory analysis. However, the cost-effectiveness assessment shows that PSURV-SSC remains the most cost-effective SSC of the Sylvatub system in these departments, because of its high sensitivity and because of the higher number of animals processed [10]. Coordination and collection activities are particularly costly for the PSURV-SSC, because of regular information and awareness-raising meetings, in order to present the sampling objectives, the geographical areas targeted and the role of each actor. However, PSURV-SSC of wild boars and red deer in high risk-level areas seems to be the least expensive SSC at individual level: this may be explained by the fact that collection costs are lower than for other SSC (no compensation for the collection) and lower than for badgers (which requires in addition the supervision of trapping in the field for the coordination category and higher costs of specific equipment for the collection category).

Cost-effectiveness should be interpreted together with CSe and total cost [11]. For example, PSURV-SSC was the most cost-effective SSC, but it requires a relatively large investment (Table 4) and is not representative of all areas. These results are only indicative for one hunting season, because variations of the epidemiological situation influence the level of risk, and thus the SSC applied and the financial agreements between partners. Furthermore, there is a strong relation between sample size, costs and sensitivity, so cost-effectiveness of a same SSC could vary depending on the number of collected animals (itself dependent on the local epidemiological situation and the level of sampling intensity) [11].

Thus, PSURV-SSC seemed to be highly sensitive and cost-effective in high-risk level areas. EC-SSC was also sensitive and reasonably cost-effective and it allows the detection of bTB in all areas, regardless the risk-level, but is not applied in badgers. SAGIR-SSC had a low probability of detection and a low cost-effectiveness, due to the small number of animals collected for the amount of expenditure. The recommendations for veterinary authorities could be (i) to maintain PSURV-SSC in infected areas, even if it requires an important financial investment, (ii) to reinforce the EC-SSC in all areas (low-, medium- and high-risk), through information campaigns and hunters’ training to the detection of TBLs, given its moderate costs and correct sensitivity, (iii) to keep a normal functioning of the SAGIR-SSC in all areas, which could provide a continuous surveillance even in summer on all species, without any reinforcement for the Sylvatub system as it is not clearly cost-effective.

### 4.3 Perspectives

This study allowed to assess current surveillance components in France. However, it would be interesting to evaluate the effectiveness and cost-effectiveness of alternative prospective surveillance strategies [19]. Different hypothetical scenarios could be evaluated and compared to the current surveillance system before being implemented [19; 23].

Furthermore, the indirect costs of the implementation of management measures (reduction of densities, destruction of animal viscera, etc.) could have an impact on the global economic analysis (costs and benefit) regarding bTB (including surveillance and control) and on the acceptability of the measures by the actors. Thus, the indirect impact of the bTB Sylvatub surveillance could also be considered in terms of global epidemiological and economical benefit to cattle population, trade facilitation and public health [14, 24, 25, 26, 27, 28]. In fact, with an effective surveillance in wildlife, adapted control measures could be applied, which in turns reduce the prevalence in wildlife and thus decrease the probability of transmission to cattle. Such more complex approach on the evaluation of the impact of the bTB wildlife surveillance on cattle situation (at a national or international level) require more complex models such as computer general equilibrium models and/or the use of social accounting matrix [14].

Finally, the inclusion of behavioural effects (the hunters’ awareness of the disease, their willingness to report a suspicion, their acceptability of the surveillance and control measures, etc.) are important for the evaluation of the performance and of the costs of the system, and therefore for decision-making. In fact, if a SSC is more costly for hunters, they could disengage themselves for the surveillance activity, which could in return induce a decrease of the effectiveness of the surveillance [26]. Investigations are performed to identify the key factors which influence the commitment of stakeholders in each SSC, by level of risk.

In conclusion, this was the first economic analysis of bTB surveillance in wildlife in France. We conclude that PSURV-SSC seemed to be highly sensitive and cost-effective in high-risk level areas. EC-SSC was also sensitive and reasonably cost-effective and it allows the detection of bTB in all areas, regardless the risk-level, but is not applied in badgers. SAGIR-SSC had a low probability of detection and a low cost-effectiveness, due to the small number of animals collected for the amount of expenditure. Further investigations should be performed to compare actual and potential alternatives surveillance strategies in order to optimize the allocation of financial and human resources for bTB management in wildlife.

## Supporting information

S1 Fig Scenario tree illustrating the scanning surveillance system component based on carcass examination for hunted wild boar, red deer and roe deer (EC-SSC, applied in areas of all risk levels). (TIF)

S2 Fig Scenario tree illustrating the surveillance system component for animals found dead, moribund or with abnormal behaviour (SAGIR-SSC, applied to each species and all risk levels). (TIF)

S3 Fig Scenario tree illustrating the active surveillance system component for badger and wild boar in medium- and high-risk areas (PSURV-SSC). (TIF)
