# Supplementary figures and images for: Cost-effectiveness evaluation of bovine tuberculosis surveillance in wildlife in France (Sylvatub system) using scenario trees
Source: PLoS One. 2017 Aug 11;12(8):e0183126. doi: 10.1371/journal.pone.0183126 (PMC5553909; doi:10.1371/journal.pone.0183126)

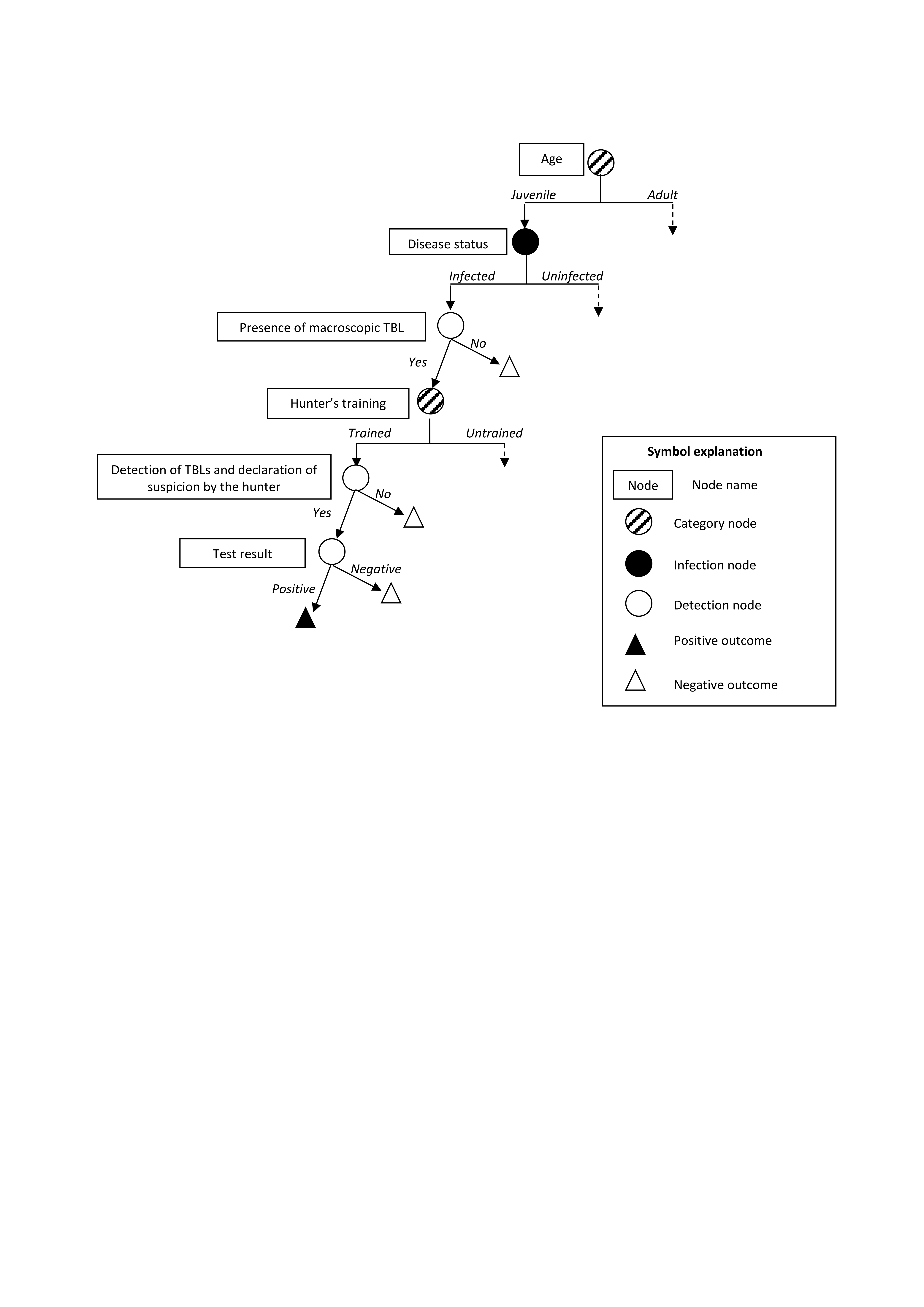

Supplement: S1 Fig — (TIF) [file pone.0183126.s001.tif]

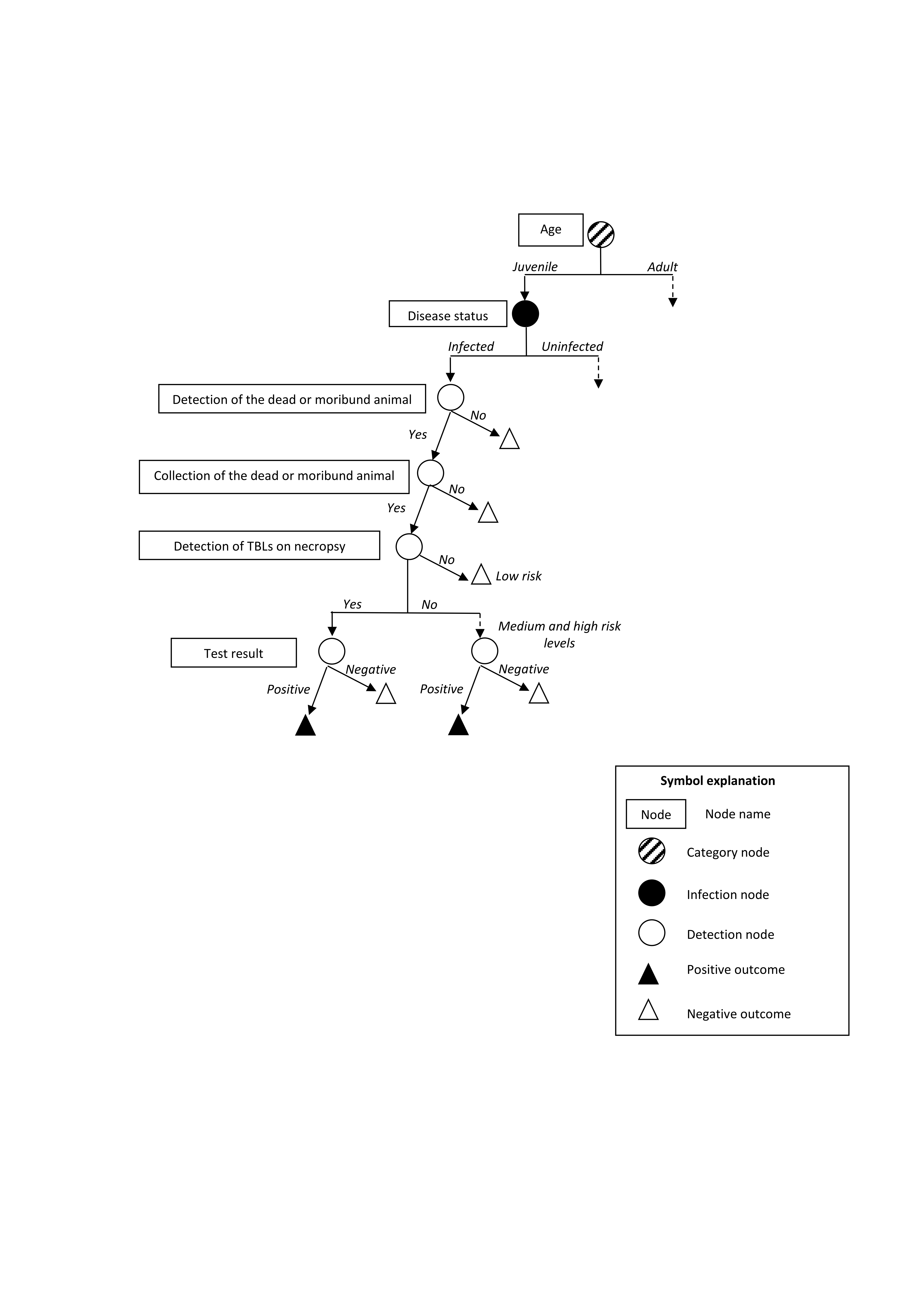

Supplement: S2 Fig — (TIF) [file pone.0183126.s002.tif]

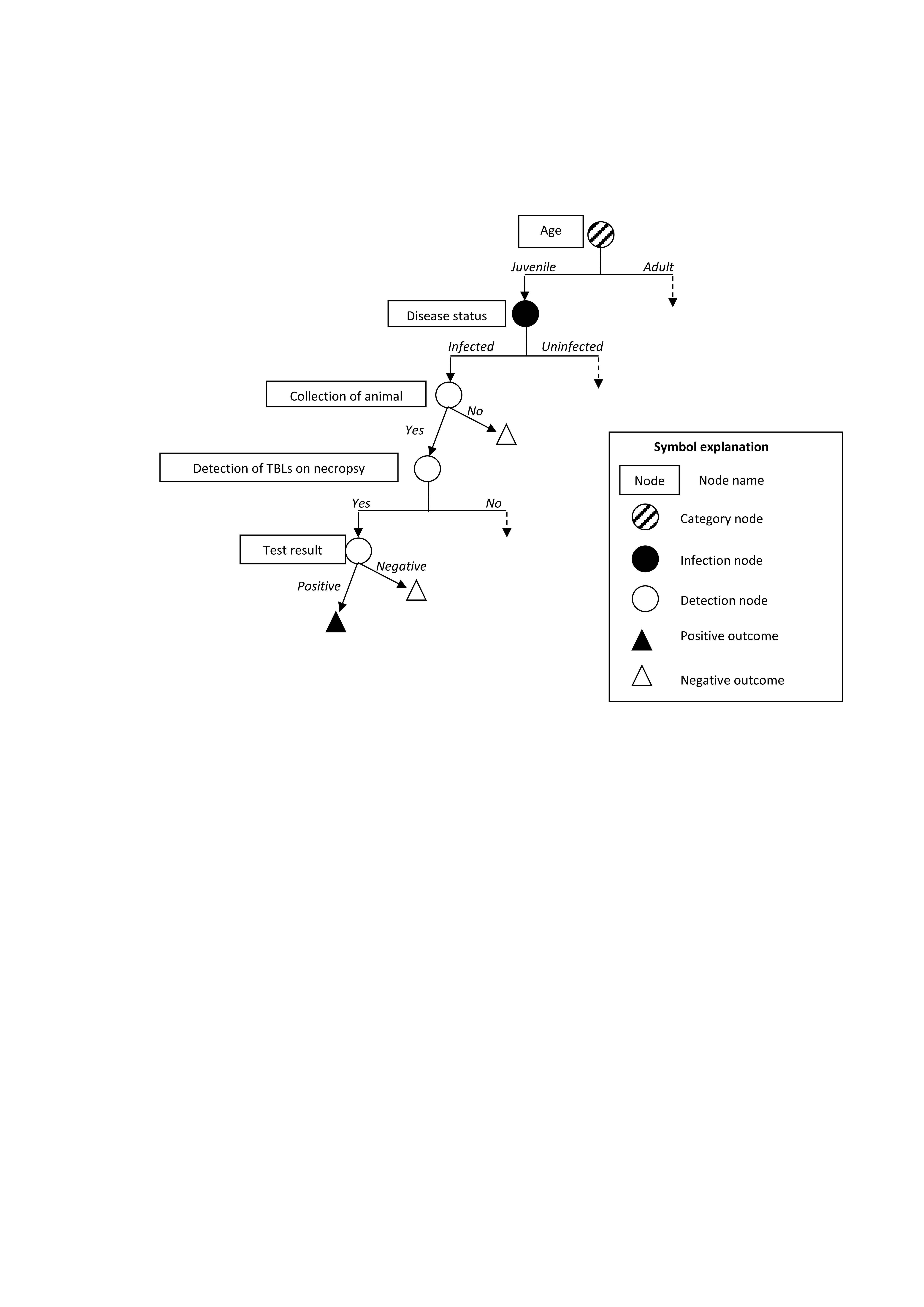

Supplement: S3 Fig — (TIF) [file pone.0183126.s003.tif]
